# Pathways to Upscaling Highly Efficient Organic Solar Cells Using Green Solvents: A Study on Device Photophysics in the Transition from Lab‐to‐Fab

**DOI:** 10.1002/advs.202402637

**Published:** 2024-06-17

**Authors:** Eva Mazzolini, Richard A. Pacalaj, Yuang Fu, Bhushan R. Patil, Rahul Patidar, Xinhui Lu, Trystan M. Watson, James R. Durrant, Zhe Li, Nicola Gasparini

**Affiliations:** ^1^ School of Engineering and Materials Science (SEMS) Queen Mary University of London London E1 4NS UK; ^2^ Department of Chemistry & Centre for Processable Electronics Imperial College London London W12 0BZ UK; ^3^ Department of Physics The Chinese University of Hong Kong New Territories Hong Kong SAR 999077 China; ^4^ SPECIFIC College of Engineering Swansea University Bay Campus Swansea SA1 8EN UK

**Keywords:** green solvents, OPV, recombination, transient photovoltage, upscaling

## Abstract

As the rise of nonfullerene acceptors (NFA) has allowed lab‐scale organic solar cells (OSC) to reach 20% efficiency, translating these devices into roll‐to‐roll compatible fabrication still poses many challenges for researchers. Among these are the use of green solvent solubility for large‐scale manufacture, roll‐to‐roll compatible fabrication, and, not least, information on charge carrier dynamics in each upscaling step, to further understand the gap in performance. In this work, the reproducibility of champion devices using slot‐die coating with 14% power conversion efficiency (PCE) is demonstrated, under the condition that the optimal thickness is maintained. It is further shown that for the donor:acceptor (D:A) blend PM6:Y12, the processing solvent has a more significant impact on charge carrier dynamics compared to the deposition technique. It is found that the devices processed with o‐xylene feature a 40% decrease in the bimolecular recombination coefficient compared to those processed with CB, as well as a 70% increase in effective mobility. Finally, it is highlighted that blade‐coating yields devices with similar carrier dynamics to slot‐die coating, making it the optimal choice for lab‐scale optimization with no significant loss in translation toward up‐scale.

## Graphical abstract

Here, organic photovoltaics using slot‐die coating deposition method and green solvents delivering power conversion efficiencies of 14% are shown. It is found that the processing solvent has a more significant impact on charge carrier dynamics compared to the deposition technique. It is highlighted that blade‐coating yields similar carrier dynamics to slot‐die coating, making it the optimal choice for lab‐scale optimization.

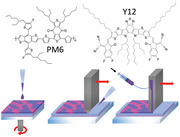

## 1 Introduction

Since the development of nonfullerene acceptors, organic solar cells (OSCs) have made strides toward reaching to 20% power conversion efficiency (PCE) in just a few years.^[^ ^1^ ^]^ Their potential in applications such as the Internet of Things,^[^ ^2^ ^]^ building integrated photovoltaics,^[^ ^3^ ^]^ and agrivoltaics,^[^ ^4^ ^]^ has pushed researchers to make significant progress in terms of performance and stability, however, devices and large area modules made with upscaling‐friendly techniques are still lagging.^[^ ^5^ ^]^

Suffering from many losses related to, but not limited to, sheet resistances of large‐area electrodes, active layer morphology variations, and thickness limitations, upscaled devices still need significant efforts from researchers to achieve performances comparable to their lab‐scale counterparts.^[^ ^6^, ^7^, ^8^ ^]^

Active layer processing is generally thought to have a drastic impact on device performance when going from lab‐scale techniques such as spin‐coating to roll‐to‐roll compatible ones such as slot‐die coating.^[^ ^5^ ^]^ Currently, the literature has mainly reported OSCs with active layers deposited as highly reproducible spin‐coated films, with an optimal bulk heterojunction morphology for high performance, on small‐area pixels. However, a direct translation of these champion devices into up‐scalable ones is not straightforward.^[^ ^9^ ^]^ This is because the morphology of active layers can change significantly when using a roll‐to‐roll friendly technique like slot‐die coating due to challenges in controlling drying kinetics and solution flow and, not least, fabrication in air most of the time, which can add constraints to materials in terms of environmental stability.^[^ ^10^ ^]^

Solvent toxicity is another significant upscaling challenge. To this day, most organic photovoltaics (OPV) research has been reporting devices made using halogenated solvents such as chloroform (CF) and chlorobenzene (CB), with some research focusing on greener alternatives.^[^ ^4^, ^11^, ^12^, ^13^, ^14^ ^]^ Recently, Luo et al. demonstrated an impressive 19% PCE using the blend D18‐Cl:L8‐BO‐X in toluene,^[^ ^15^ ^]^ reaching that of state‐of‐the‐art, CF‐processed devices.^[^ ^16^ ^]^

In the context of upscaling, it is paramount to consider that solvents have industrial limitations in many countries due to environmental and human health reasons.^[^ ^17^ ^]^ Therefore, the replacement of halogenated solvents with less toxic and more sustainable alternatives is desirable for large‐scale commercialization. Herein, the term “green solvent” refers to nonhalogenated solvents. However, the solubility of commonly reported OPV materials tends to be lower in these greener solvents. Often, this requires the use of structural analogs with tailored solubilizing side chains and solvent additives.^[^ ^18^, ^19^, ^20^ ^]^ While the switch to a green solvent seems straightforward assuming good solubility of the materials, processing solutions with different boiling points (due to the solvent itself, or any additives) can lead to active layers with drastically different morphology, which can pose challenges in optimization.^[^ ^21^, ^22^, ^23^, ^24^ ^]^

Adding to these challenges is a general lack of understanding of the impact of scaling deposition on charge carrier kinetics. Although significant efforts have been made toward understanding the active layer morphology,^[^ ^25^, ^26^, ^27^ ^]^ thickness dependence,^[^ ^28^, ^29^ ^]^ and improving device performance of large‐scale devices,^[^ ^30^, ^31^ ^]^ it is still unclear how the steps needed to upscale devices (including, but not limited to, green solvents and large‐scale friendly fabrication techniques) impact their charge carrier dynamics. Among these techniques, the most commonly used for large‐area devices are blade coating and slot‐die coating, which, at present, have been used for the fabrication of devices with PCEs of 16.1% and 15.6%, respectively,^[^ ^19^, ^32^ ^]^ showing that with thorough optimization and careful choice of materials, these deposition methods can yield very promising results.

Studying charge carrier densities, lifetimes, and mobilities, is therefore crucial to understanding performance limitations of up‐scaled devices and allows for more targeted optimization, effectively aiding in closing the performance gap between lab‐scale and large‐scale devices. While the effect of device thickness on recombination has been investigated in the past,^[^ ^33^ ^]^ as well as the impact of solvent alone,^[^ ^34^, ^35^ ^]^ these studies are generally limited to one technique only and do not compare a wide range of deposition methods. One study by Ro et al. compared spin‐coating, blade‐coating, and slot‐die coating in terms of morphology, but did not address changes in carrier dynamics.^[^ ^36^ ^]^ The motivation of this work is to shed light on the behavior of charge carriers through a side‐by‐side comparison of devices fabricated with different solvents and deposition techniques, ranging from small‐scale, lab‐friendly to large‐scale, roll‐to‐roll friendly.

In this work, we investigate the fundamental differences in device physics in two cases. We use the D:A blend PM6:Y12 to fabricate spin‐ and blade‐coated devices with o‐xylene and CB, to disentangle the effects of solvent and processing methods from the perspective of device performance, morphology, and charge carrier dynamics. We observe that CB yields higher V_oc_, which is due to an increased effective electronic bandgap as determined by Charge Extraction (CE) measurements. However, Transient photovoltage (TPV) measurements show that recombination rates are lower in o‐xylene compared to CB‐based spin‐coated and blade‐coated devices. We then study the impact of active layer deposition methods on devices made using o‐xylene, to gain insights into the potential origins of performance losses in the transition to large‐scale. Our findings suggest that the charge carrier dynamics are not strongly impacted by the fabrication technique, however, the morphology of the films is impacted by drying, as shown by Grazing‐Incidence Wide‐Angle X‐ray Scattering (GIWAXS). This is, however, not reflected in device performance, which is maintained for thin (≈100 nm) active layer devices, regardless of fabrication.

Ultimately, this work demonstrates that for PM6:Y12, the choice of solvent has a greater impact on charge carrier dynamics compared to the fabrication technique, and that optimal device performance can be maintained using slot‐die coating by maintaining thin active layers. We further show that blade‐coated devices have similar charge carrier dynamics behavior to those processed with slot‐die coating, making lab‐scale optimization directly translatable into large‐scale. Finally, we stress that slot‐die coating thin devices still pose a challenge for the community due to film homogeneity and that closing the efficiency gap requires thickness‐independent materials.

## 2 Results and Discussion

### 2.1 Solvent Comparison

**Figure 1:**
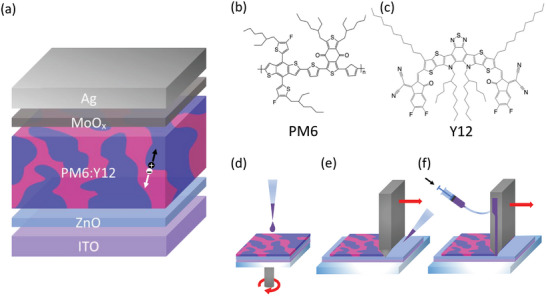
a) Device architecture, b) Chemical structure of polymer donor PM6 and c) nonfullerene acceptor Y12; Schematics of d) Spin coating, e) Blade coating f) Slot‐die coating.

As mentioned above, the transition to large‐scale requires materials soluble in green solvents. We selected state‐of‐the‐art donor and acceptor materials based on polymer donor PM6 and small molecule acceptor Y12. Y12 was chosen for its solubility in green solvents.^[^ ^19^ ^]^ The chemical structures of these materials are reported in **Figure** **1**b,c.

We fabricated organic solar cells in an inverted architecture (Figure 1a), composed of ITO on glass, ZnO electron transport layer, active layer, deposited with the three different techniques, Molybdenum oxide hole transport layer and an evaporated silver electrode (ITO/ZnO/PM6:Y12/MoO_3_/Ag).

The active layer materials were dissolved in CB and o‐xylene, as halogenated and green solvents, respectively. The two solvents were selected for their similar boiling points, aiding optimization, and for the high solubility of PM6:Y12 in both.

**Figure 2:**
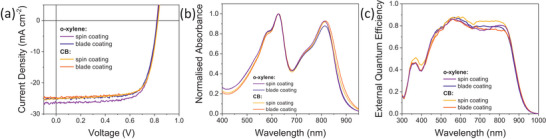
a) *J–V* characteristic of highest performing devices using o‐xylene and chlorobenzene, b) Corresponding normalized absorbance spectra, c) External quantum efficiencies of champion devices.

PM6:Y12 devices in CB and o‐xylene were first fabricated using a spin coating (Figure 1d), with an optimal active layer thickness of ≈100 nm.^[^ ^19^ ^]^ Their current‐voltage characteristics as well as their photovoltaic performance are summarised in **Figure** **2** and **Table** **1**, respectively.

**Table 1:** Highest‐performing devices, average (min. 8 devices), and standard deviation of photovoltaic parameters of devices made using o‐xylene and CB, measured under AM 1.5G illumination.

Solvent	Technique	PCE [%]	V_oc_ [V]	FF	J_sc_ [mA cm^−2^]
o‐xylene	spin coating	15.5 (14.6 ± 0.5)	0.817 (0.815 ± 0.002)	0.72 (0.71 ± 0.02)	26.47 (25.23 ± 0.88)
	blade coating	15.6 (14.5 ± 0.8)	0.823 (0.820 ± 0.004)	0.75 (0.74 ± 0.01)	25.34 (23.98 ± 1.30)
CB	spin coating	15.4 (15.0 ± 0.4)	0.828 (0.825 ± 0.002)	0.74 (0.73 ± 0.01)	25.09 (25.02 ± 0.76)
	blade coating	15.4 (14.8 ± 0.6)	0.835 (0.831 ± 0.003)	0.75 (0.74 ± 0.01)	24.70 (23.91 ± 0.96)

Interestingly, the o‐xylene devices maintained similar PCE compared to CB, going from an average of 15.0–14.6% in the green solvent. This was also reflected in the short circuit current density (J_sc_), whose values remained similar, with an average of 25.02 mA cm^−2^ in CB and 25.23 mA cm^−2^ for o‐xylene. The average fill factor (FF) values also remained similar, from 0.73 in CB to 0.71 in o‐xylene. The only photovoltaic parameter that suffered from losses was the open circuit voltage (V_oc_), which, on average, decreased by ≈10 mV with o‐xylene.

To further explore these results, the devices were replicated using blade coating (Figure 1e), with a bed temperature of 80 °C and an active layer thickness of 100 nm. Although the two techniques have significantly different film drying times, the performance of the blade‐coated devices was comparable to their spin‐coated counterparts, and the photovoltaic parameters followed the same trends. Specifically, the average PCE went from 14.8% to 14.6% from CB to o‐xylene, the J_sc_ increased from 23.91 to 23.98 mA cm^−2^ with o‐xylene, and a 10 mV decrease in average V_oc_ was observed again (Table 1).

The comparison between spin‐coated and blade‐coated devices is also noteworthy: using o‐xylene as a benchmark solvent, we can conclude that high performance is achievable with blade coating for PM6:Y12 (maximum PCE of 15.6% compared to 15.5% for spin‐coating), with no loss in V_oc_, higher average FF (0.74 with blade coating compared to 0.71 with spin coating), and a loss in average J_sc_ of ≈1.2 mA cm^−2^ (Table 1).

Importantly, a similar loss in J_sc_ was also found for CB, with its average value decreasing from 25.02 mA cm^−2^ for spin‐coated devices to 23.98 mA cm^−2^ for blade‐coated ones.

These results suggest that for the PM6:Y12 blend, performance can be maintained with both solvents at similar thicknesses. However, when looking at the maximum achievable V_oc_ for CB and o‐xylene, it is notable that CB devices reach 0.83 and 0.84 V with spin‐ and blade‐coating respectively, while o‐xylene devices are limited at 0.82 V with both fabrication techniques. This loss is significantly lower than solvent‐dependent V_oc_ changes observed in PM6:Y6 when switching from chloroform to chlorobenzene or o‐xylene (ΔV_oc_ > 50 mV),^[^ ^37^, ^38^ ^]^ highlighting the advantages of Y12's improved solubility. We have calculated Flory−Huggins interaction parameter, χ, from the contact angle measurements of the donor and acceptor materials processed with different solvents and methods. **χ ** is calculated as **χ ∝ (√ 𝛾_1_ – √ 𝛾_2_)^2^**, with **𝛾_1_** and **𝛾_2_** as the surface free energies (SFE) of the single materials). The χ values extracted range between 0.1 and 0.2, indicating well‐mixed phases between two components independent of solvent and fabrication (Table S1, Supporting Information).^[^ ^39^, ^40^, ^41^ ^]^

In the following, the small loss in V_oc_ was investigated using charge extraction and transient photovoltage measurements.

**Figure 3:**
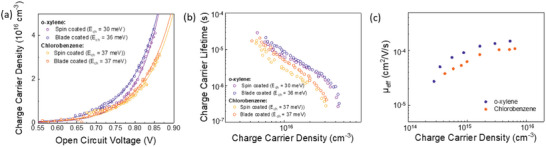
a) Charge carrier densities of devices fabricated with o‐xylene and CB using spin‐coating and blade coating, measured using charge extraction, b) Corresponding charge carrier lifetimes measured using transient photovoltage, c) Effective mobilities of spin‐coated devices with o‐xylene and CB, measured using transient photovoltage.

**Figure** **3**a shows the charge density quantified by charge extraction measurements versus the open circuit voltage (used as a proxy for the quasi‐Fermi‐level‐splitting). At equivalent voltages, the active layers produced by o‐xylene show an increase of the spatially averaged charge density and a slightly reduced effective electronic gap in line with the changes in V_oc_. No change in the optical gap of the acceptor material was discernible (Figure 2c). Therefore, we assign changes in the effective gap to interfacial electrostatic effects as previously observed in closely related Y6 blends.^[^ ^37^ ^]^ Despite the difference in drying kinetics of the wet film for spin‐coated versus blade‐coated devices, the charge carrier density built‐up seems more affected by the choice of solvents. Fitting the charge density data using the equation $n\;\left(V_{\mathrm{oc}}\right)=n_{0}\;\exp\left(\frac{eV_{OC}}{2E_{\mathrm{ch}}}\right)$ (where *n* is the charge carrier density as a function of the V_oc_, *n_0_* is a fitting parameter corresponding to the intrinsic charge density and *e* is the electron charge)^[^ ^42^, ^43^ ^]^ yields only minor differences in the characteristic energy (*E*_ch_) of the exponential density of tailstates. Only the device fabricated by spin‐coating from o‐xylene exhibits a narrower bandtail signified by a reduced *E*_ch_ (30 meV vs 36 meV for the blade‐coated device). The observed values are similar to those previously reported for the closely related acceptor Y6.^[^ ^42^, ^44^ ^]^ Transient photovoltage measurements reveal a similar dependence of the charge carrier lifetime on the *V*_oc_ (Figure S1, Supporting Information) for all four devices. When combining the charge carrier lifetimes with the spatially averaged charge density data, it becomes apparent that the nongeminate recombination is slowed down in the o‐xylene devices at equivalent charge carrier density values. At a charge density of 2 × 10^16^ cm^−3^, similar to the value observed at 1 sun *V*_oc_ conditions, the bimolecular recombination coefficients *k*_BI_ (assuming a second order process) were calculated to be 2.8 × 10^−11^ and 1.75 × 10^−11^ cm^3^s^−1^ for the o‐xylene and CB devices, respectively.

Measurements of the steady‐state charge carrier density in the active layer at short circuit conditions were used to calculate the effective mobility for the devices spin‐coated from o‐xylene and chlorobenzene following the method described by Shuttle et al.^[^ ^45^ ^]^ (see Figure 3c). The effective mobility shows a slight increase for o‐xylene compared to the CB device (1.7 vs 1.0 × 10^−4^ cm^2^ V^−1^ s^−1^ measured at a charge density of 2 × 10^16^ cm^−3^) while the mobility dependence on the charge carrier density remains unchanged. The increase of the effective mobility by 70% combined with a decrease of the bimolecular recombination coefficient by ≈40% for the o‐xylene device results in an overall improved Langevin reduction factor of 8.2 versus 3.2 for CB. The electronic quality factor *Q* ^[^ ^46^ ^]^ improves from 3.0 to 11.6. Similar observations of reduced recombination rate constants and increased mobility values have previously been made for PM6:Y6 blends deposited from CF with chloronaphtalene as a solvent additive that resulted in increased crystallinity and a reduction in energetic disorder.^[^ ^47^, ^48^ ^]^ In both cases, the observation of an increased charge carrier density accumulated in the devices at equivalent values of *V*_oc_ while observing no discernible change in the observed lifetimes may be linked to morphological changes assessed below.

**Figure 4:**
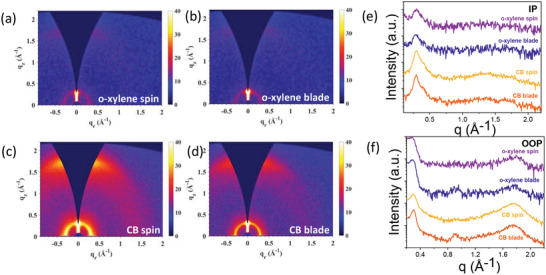
2D GIWAXS scattering patterns for a) spin‐coated o‐xylene devices, b) blade‐coated o‐xylene devices, c) spin‐coated CB devices, d) blade‐coated CB devices, e) corresponding 1D plots in the in‐plane direction and f) out‐of‐plane direction.

To further understand the impact of solvents on the active layer's microstructure, Grazing Incidence Wide‐Angle X‐ray Scattering (GIWAXS) was performed on the CB and o‐xylene films. The 2D and 1D plots are shown in **Figure** **4**, while the in‐plane and out‐of‐plane crystalline coherence lengths (*L*_c_) are summarized in **Table** **2**.

**Table 2:** Crystalline coherence lengths in the in‐plane and out‐of‐plane directions extracted from GIWAXS measurements.

Direction	Sample	Peak 1		L_c_ [Å] from Peak 1	Peak 2	
		q [Å^−1^]	d [Å]		q [Å^−1^]	D
IP	o‐xylene spin	0.30	21.0	37.9		
	o‐xylene blade	0.29	21.6	55.9		
	CB spin	0.29	21.4	69.9	0.39	16.2
	CB blade	0.29	21.6	83.7	0.38	16.7
OOP	o‐xylene spin	0.28	22.4	n/a		
	o‐xylene blade	0.29	21.7			
	CB spin	0.30	20.9	23.1	1.76	3.57
	CB blade	0.31	20.3	24.1	1.76	3.57

All blend films exhibit a (100) peak at 0.29 Å along both in‐plane (IP) and out‐of‐plane (OOP) directions with a (010) peak along the OOP directions, suggesting molecules mainly adapt to the face‐on orientation, consistent with previous studies.^[^ ^19^ ^]^ The in‐plane peak at ≈0.29 Å involves contributions from both the backbone‐on packings of Y12 and lamellar packings of PM6.^[^ ^49^, ^50^ ^]^ As shown in Table 2, the crystalline coherence lengths (*L*_c_) associated with this peak are higher in the blade‐coated devices compared to the spin‐coated devices for both solvents used. Additionally, extra peaks at ≈0.9 Å appear along the out‐of‐plane (OOP) direction only for blade‐coated devices, which could be assigned to the (300) peak of PM6 lamellar packings.^[^ ^50^ ^]^ The enhanced crystal packing may be accompanied by excessive phase segregation, compromising the exciton dissociation in the spectral region of Y12 (Figure 2c), which could explain the overall lower *J_sc_* in blade‐coated devices compared to the spin‐coated devices for both solvents. This observation is also confirmed by Atomic Force Microscopy (AFM) measurements (Figures S3–S5, Supporting Information). Blade‐coated PM6:Y12 films show a higher degree of phase separation compared to their spin‐coated counterparts, independent of the solvent used.^[^ ^51^ ^]^

Having established o‐xylene as a suitable upscaling solvent, the study of the effect of the deposition method on the optoelectronic characteristics and device performance is extended to slot‐die‐coated devices in the next section.

### 2.2 Fabrication Comparison in O‐Xylene

**Figure 5:**
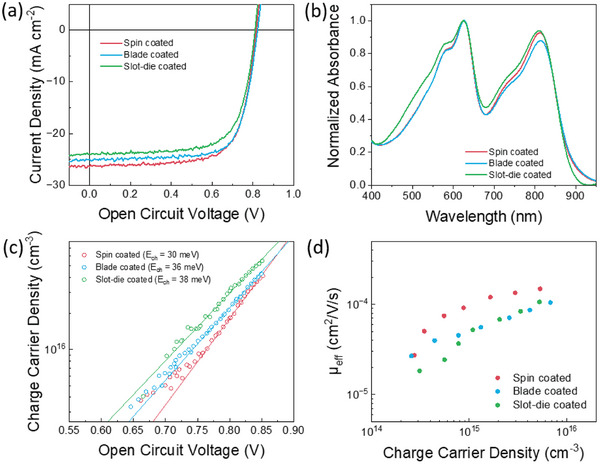
a) Current‐Voltage characteristics of 100 nm PM6:Y12 devices fabricated with different deposition techniques using o‐xylene, b) Corresponding normalized absorbance spectra, c) Charge carrier density of devices with different active layer fabrication measured using Charge Extraction, d) Effective mobility of devices with different active layer fabrication measured using Transient Photovoltage.

To address the gap in performance between lab‐scale and large‐scale, spin coating, blade coating, and slot‐die coating (Figure 1f) were used to deposit PM6:Y12 and produce devices with the architecture in Figure 1a using o‐xylene. Their J‐V characteristics under 1 sun illumination, normalized absorbance, as well as charge carrier densities and effective mobilities are shown in **Figure** **5**. The performance of these devices is reported in **Table** **3** as a function of active layer thickness.

**Table 3:** Photovoltaic performance of champion o‐xylene devices fabricated with spin coating, blade coating, and slot‐die coating, as a function of active layer thickness, measured under AM 1.5G illumination.

Active layer thickness	Coating type	PCE [%]	V_oc_ [V]	FF	J_sc_ [mA cm^−2^]
Thin	Spin coating	15.5	0.82	0.72	26.47
(≈100 nm)	Blade coating	15.6	0.82	0.75	25.34
	Slot‐die coating	13.9	0.81	0.73	23.60
Thick	Spin coating	14.7	0.81	0.73	24.84
(>150 nm)	Blade coating	14.8	0.81	0.69	26.58
	Slot‐die coating	12.0	0.81	0.63	23.50

Interestingly, the 100 nm slot‐die coated devices only suffered a 10% reduction in PCE and *J_sc_* compared to their spin‐coated equivalents, no losses in V_oc_, and maintained a high fill factor of 0.73. Strikingly, these devices reached a high PCE of 13.9%, with a V_oc_ of 0.81 V, and a *J_sc_* of 23.60 mA cm^−2^.

However, it is important to highlight the fabrication issues that are reflected in the photovoltaic performance of thicker devices for all three techniques. In fact, as the samples become thermally dried (blade coating, slot‐die coating), the active layer films suffered from thickness gradients and inhomogeneities, yielding lower fill factors at higher thicknesses compared to their spin‐coated counterparts (Table 3). In fact, the fill factor in thick devices remained high in the spin‐coated devices (0.73), suffered a loss from 0.75 to 0.69 in blade coating, and a more significant loss in slot‐die, going from 0.73 to 0.63. As such, it is fundamental to acknowledge that whilst high performance can be achieved with thin films made with slot‐die coating, reaching optimal control of film homogeneity and reproducibility are key priorities for optimization using this method.

As discussed above, a shift in the effective electronic bandgap was observed when changing from CB to o‐xylene (for spin‐coated and blade‐coated devices). Extending the optoelectronic characterizations for spin‐ and blade‐coated devices to slot‐die‐coated devices, a similar increase of the charge carrier density (at a given *V*_oc_) compared to devices produced with spin‐ and blade‐coating is observed in the slot‐die coated device. For better comparability, the chosen devices possessed similar active layer thicknesses. Looking in more detail at the exponential fits of the charge density versus *V*_oc_ data, a further small increase in energetic disorder is observed for the slot‐die device (30 meV vs 36 meV vs 38 meV for spin‐, blade‐ and slot‐die coated devices, respectively). Figure 5c shows the results on a log scale to better emphasize the differences in the exponential tailslopes. At the same time, a small reduction in the effective mobility (≈30%) is observed for active layers deposited by blade‐ and slot‐die coating.

It is apparent that the mobility as well as the charge accumulation behavior of blade‐ and slot‐die coating are clustered together. The observed behavior is in line with the thermal drying kinetics of the blade‐ and slot‐die films compared to the dynamically dried spin‐coated films. Overall, the solvent choice still outweighs the deposition method as discussed above. The results also indicate that blade‐coating may be a facile laboratory‐scale technique to investigate the upscale potential of materials without the high material demand of slot‐die coating.

The absorption spectra in Figure 5b do not show significant differences between the three techniques, suggesting that a thorough optimization, as well as maintaining the same thickness, are sufficient to avoid drastic morphological changes during upscaling for these materials.

**Figure 6:**
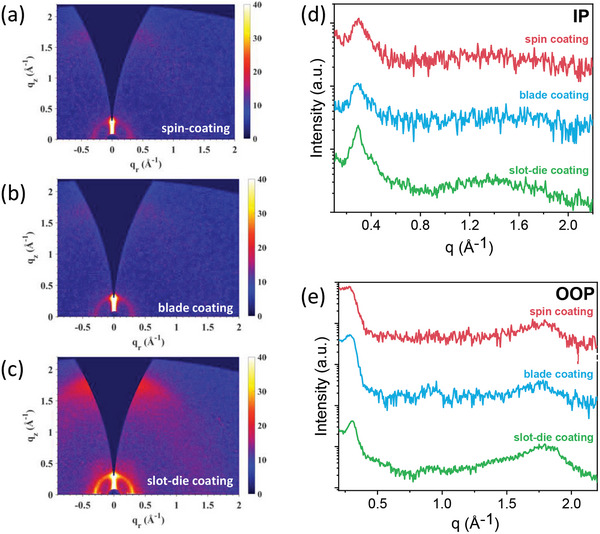
2D GIWAXS scattering patterns for o‐xylene devices fabricated with a) spin coating, b) blade coating, c) slot‐die coating, and d) corresponding 1D plots in the in‐plane direction and e) out‐of‐plane direction.

The microstructure of the films was probed using GIWAXS, and their 2D and 1D scattering patterns are shown in **Figure** **6**, while their corresponding in‐plane and out‐of‐plane crystalline coherence lengths are summarized in **Table** **4**.

**Table 4:** Crystalline coherence lengths of upscaled devices processed with o‐xylene, extracted from GIWAXS measurements.

Direction	Sample	Peak 1		L_c_ [Å] from Peak 1	Peak 2	
		q [Å^−1^]	d [Å]		q [Å^−1^]	d
IP	Spin coated	0.30	21.0	37.9		
	Blade coated	0.29	21.6	55.9		
	Slot‐die coated	0.29	21.8	59.5	0.41	15.3
OOP	Spin coated	0.28	22.4	n/a		
	Blade coated	0.29	21.7	n/a		
	Slot‐die coated	0.31	20.3	24.1	1.80	3.49

We found that the degree of crystallinity increases progressively from spin‐coated to blade‐coated to slot‐die‐coated devices, as evidenced by the stronger GIWAXS scattering intensity and larger crystal coherence lengths for (100) peaks (Table 4). The additional OOP peak at 0.9 Å^−1^ that was found for blade‐coated films is also apparent in slot‐die‐coated films (Figure 6e). Overall, it appears that the slower drying kinetics during blade and slot‐die coating processes may induce over‐phase segregation of blend films. Compared to dynamically‐dried spin‐coated devices, the blade and slot‐die‐coated devices with enhanced crystal packing are likely to have reduced light‐to‐current conversion, resulting in the lower *J_sc_* measured. Similar to above, a higher degree of phase separation is also observed for the slot‐die‐coated blends compared to the spin‐coated films measured using Atomic Force Microscopy (Figure S6, Supporting Information).^[^ ^51^ ^]^

## 3 Conclusion

In summary, we demonstrated that for the PM6:Y12 blend, solvents have a stronger impact on charge carrier dynamics compared to the fabrication technique as evidenced by the charge accumulation behavior and the shift in the effective electronic gap. Furthermore, we showed that losses in V_oc_ with o‐xylene compared to CB are attributed to an energetic shift and that devices processed with o‐xylene have higher effective mobilities and longer carrier lifetimes, resulting in lower recombination rates. The microstructural characterizations as well as the transient measurements suggest that solvent choice overrides the impact of drying kinetics due to the different fabrication techniques. Simultaneously, we showed that the *V*_oc_ losses due to switching solvents observed in Y12 are significantly smaller than those previously observed for Y6, demonstrating the robustness of Y12 due to its superior solubility in a range of solvents.

We then demonstrated champion devices reaching 14% PCE using slot‐die coating and a green solvent, and we showed that charge carriers behave similarly in thermally dried samples, making blade‐coating a valid optimization option in labs where sheet‐to‐sheet or roll‐to‐roll equipment is not available. By using blade coating and green solvents, we were able to test devices in a cost‐effective, nontoxic, and low‐waste manner before making the leap toward slot‐die.

We finally stressed that performance (and, specifically, FF) losses are present in slot‐die coating only when the optimal thickness is not maintained. This results in inhomogeneities in the film due to the coating and drying properties of this technique, which highlights once again the necessity for thickness‐independent materials for upscaling.

## 4 Experimental Section

### Device Fabrication

The organic solar cells were fabricated using an inverted architecture composed of ITO/ZnO/PM6:Y12/MoO_x_/Ag. Glass/ITO substrates of 12 × 12 and 25 × 25 mm were cleaned by a sequential sonication process of distilled water, acetone, and isopropanol. The substrates were then treated with oxygen plasma for 8 min.

The sol‐gel ZnO electron transport layer was made by dissolving 219.5 mg zinc acetate dehydrate in 2 mL 2‐methoxyethanol and 60.4 µL ethanolamine and stirred overnight at room temperature. The ETL was then spin‐coated on the ITO substrates at 4000 rpm for 40 s and annealed at 150 °C for 10–15 min in air.

The active layer solution was composed of PM6:Y12 in a 1:1.2 ratio and a 22 mg mL total concentration. The materials were dissolved in anhydrous chlorobenzene or o‐xylene in a nitrogen‐filled glovebox and the solutions were stirred overnight.

The spin‐coated active layers were deposited at 3000 rpm for CB and 3500 rpm for o‐xylene for 40 s. They were then annealed at 100 °C for 10 min.

The blade‐coated devices were made by dropping 20 µL of the solution onto the substrates with a blade speed of 10 mm ^−1^s for o‐xylene and 20 mm ^−1^s for CB to maintain a dry thickness of 100 nm (specifically, 102, 107, 100, and 103 nm for CB spin‐coated, CB blade coated, o‐xylene spin‐coated and o‐xylene blade coated, respectively). The blade height was set to 400 µm and the blade‐coater bed was set to 80 °C. The active layers were then annealed at 80 °C for 5 min in air.

The slot‐die coated devices were made using a temperature‐controlled slot‐die headset at 70 °C and the slot‐die bed was set to 80 °C. The solution was pumped with a flow rate of 0.025 mL min^−1^ and a speed of 0.5 m min^−1^. The meniscus guide height was kept at 100 µm. The films were then annealed at 80 °C for 5 min in air.

Finally, 10 nm of MoO_x_ and 100 nm of Ag were thermally evaporated with rates of 0.1 and 0.5 A s^−1^ respectively.

### Device Characterization

The current–voltage characteristics of the devices were measured using a 2400 Keithley Source‐Measure unit and an Oriel Instruments Solar Simulator AAA with a xenon lamp that was calibrated using a Newport Silicon cell to ensure AM1.5G. The devices were measured in a nitrogen‐filled chamber. Light‐intensity‐dependent measurements were conducted by placing Thorlabs ND filters with varying optical densities on top of the chamber. The EQE was measured in air with a Quantum Design PV300 system.

### Imaging

The AFM images were obtained using an Agilent Technologies Keysight 5500 SPM.

### Optical Characterization

UV–vis measurements were done on active layer films on glass using a UV‐1601 Shimadzu spectrometer.

### GIWAXS Measurements

GIWAXS characterization was done with a Xeuss 2.0 SAXS/WAXS laboratory beamline with a Cu X‐ray source (8.05 keV, 1.54 Å) and a Pilatus3R 300 K detector.

### Photophysics Characterizations

A ring of six warm and six cold LEDs was used as a variable light source to create the steady‐state open circuit conditions for the transient photovoltage and charge extraction experiments. The light intensity was calibrated to current match the *JV*‐curves measured using the solar simulator. To determine the charge carrier density at open circuit conditions, the LED background illumination was switched off and simultaneously the device was switched to short circuit conditions. MOSFETs with a switching time of 100 ns were used for this purpose and were controlled by the DAQCard which was also used to trigger the oscilloscope. The resulting current transient was recorded using a Tektronix TDS3032B oscilloscope to record the voltage drop across a 50 Ω measurement resistor and convert this to a current measurement using Ohm's law. Integration yields the total charge carrier density. To determine the active layer charge, this was corrected for the electrode charge by measuring the geometric capacitance using charge extraction from low reverse bias charging up the device electrodes.^[^ ^52^ ^]^

Lifetimes from transient photovoltage measurements were determined at equivalent open circuit conditions. Additionally, a laser pulse was used to generate a small perturbation voltage (Minilite Continuum II, 5 ns, 532 nm). The resulting voltage decay back to the steady‐state open circuit voltage was recorded using the 1 MΩ input impedance of the oscilloscope. The oscilloscope was triggered by a photodiode that records the laser light. By fitting a single exponential to the voltage decay, the small perturbation lifetime was measured that could be converted to the full signal pseudo‐first‐order lifetime using the measured recombination order as outlined elsewhere.^[^ ^53^ ^]^

For the determination of the effective mobility, the steady‐state charge carrier density at a short circuit was measured by charge extraction. The process was equivalent to the charge extraction at an open circuit, but the device was kept at a short circuit throughout. The mobility was calculated using an analytical model as outlined elsewhere.^[^ ^44^ ^]^

## Conflict of Interest

The authors declare no conflict of interest.

## Supporting information

Supporting Information

## Data Availability

The data that support the findings of this study are available from the corresponding author upon reasonable request.
