# Supplementary material for: Pathways to Upscaling Highly Efficient Organic Solar Cells Using Green Solvents: A Study on Device Photophysics in the Transition from Lab‐to‐Fab
Source: Adv Sci (Weinh). 2024 Jun 17;11(31):2402637. doi: 10.1002/advs.202402637 (PMC11336899; doi:10.1002/advs.202402637)
Supplement: Supplementary file 1 — Supporting Information [file ADVS-11-2402637-s001.docx]

**Supporting Information**

**Pathways to upscaling highly efficient organic solar cells using green solvents: a study on device photophysics in the transition from Lab-to-Fab**

Eva Mazzolini^a,b^, Richard A. Pacalaj^b^, Yuang Fu^c^, Bhushan R. Patil^d^, Rahul Patidar^d^, Xinhui Lu^c^, Trystan M. Watson^d^, James R. Durrant^b,d^, Zhe Li^a*^, Nicola Gasparini^b*^

^a^ School of Engineering and Materials Science (SEMS), Queen Mary University of London, London E1 4NS, UK

^b^ Department of Chemistry & Centre for Processable Electronics, Imperial College London, London, W12 0BZ, UK

^c^ Department of Physics, The Chinese University of Hong Kong, New Territories, Hong Kong

^d^ SPECIFIC, College of Engineering, Swansea University, Bay Campus, Swansea SA1 8EN, UK

Keywords: OPV, green solvents, upscaling, recombination, transient photovoltage

n.gasparini@imperial.ac.uk

zhe.li@qmul.ac.uk


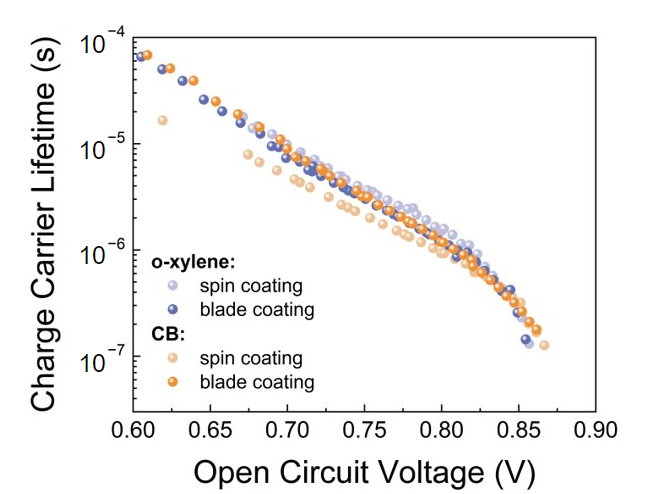


Figure S1. charge carrier lifetime extracted from TPV measurements for blade coated and spin coated devices processed from o-xylene and CB


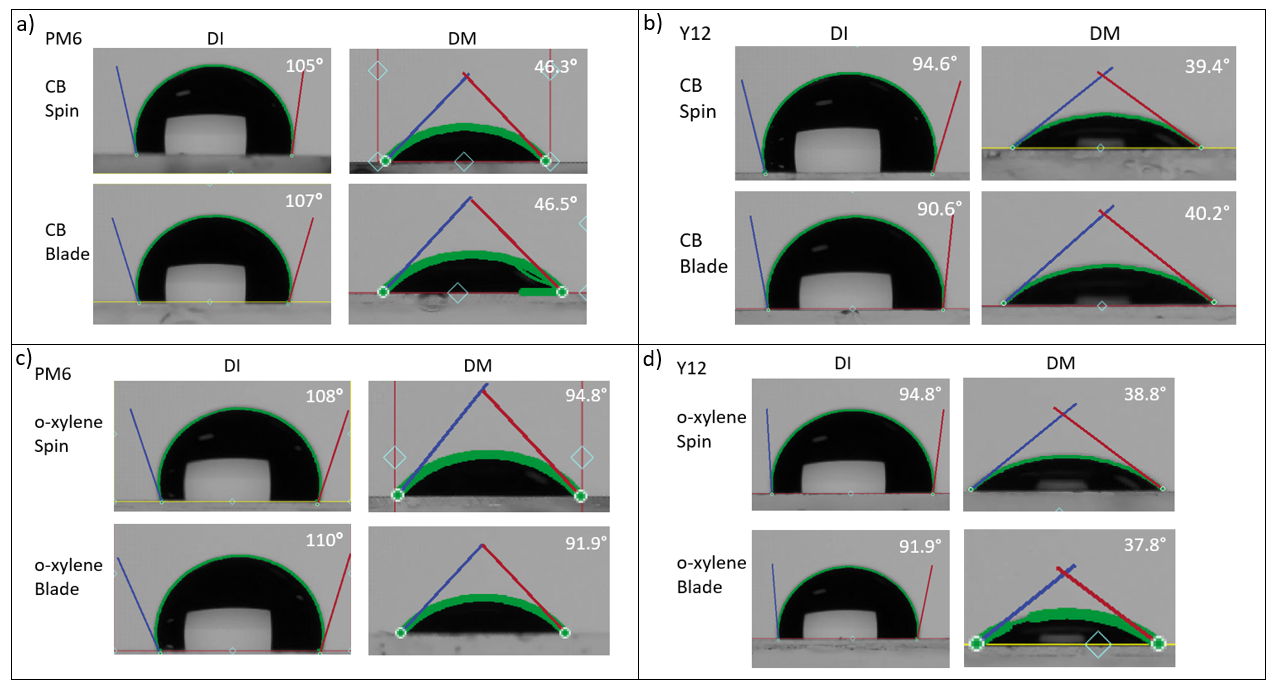

Figure S2. Contact angle measurements for PM6 and Y12, deposited from CB (a-b) and o-xylene (c-d), fabricated via spin coating and blade coating. Single droplets of de-ionized water (DI) and diiodomethane (DM) were dropped onto the thin film surface and the contact angle was recorded and analyzed. The average contact angle is reported in top right corner of the single images.

Table S1. Surface Free Energy and corresponding Flory-Huggins interaction parameter χ of neat PM6 and Y12 thin films fabricated using CB and o-xylene, deposited via spin and blade coating.

| Solvent | Fabrication | Material | SFE (mN/m) | χ |
| --- | --- | --- | --- | --- |
| CB | Spin coating | PM6 | 36.34 | 0.10 |
|  |  | Y12 | 39.93 |  |
|  | Blade coating | PM6 | 36.31 | 0.10 |
|  |  | Y12 | 40.28 |  |
| o-xylene | Spin coating | PM6 | 34.76 | 0.22 |
|  |  | Y12 | 40.51 |  |
|  | Blade coating | PM6 | 36.08 | 0.17 |
|  |  | Y12 | 41.29 |  |


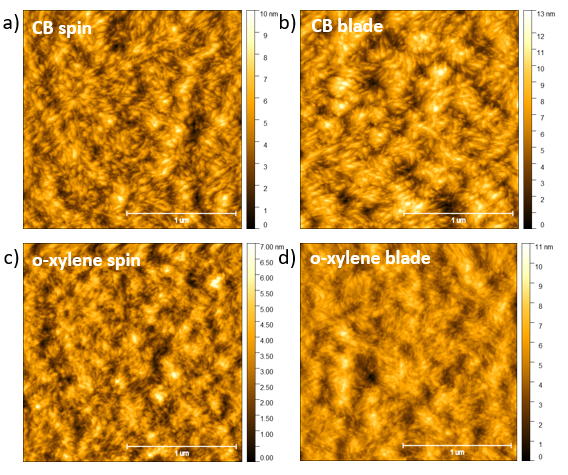


Figure S3. AFM topography images of samples processed with CB and o-xylene using a),c) spin-coating and b),d) blade coating, respectively.


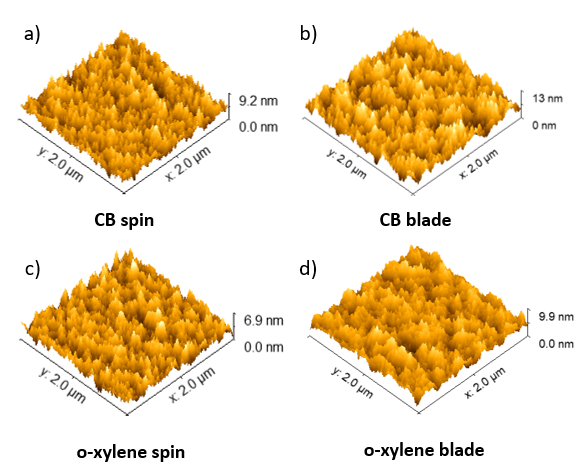


Figure S4. AFM 3D topography images of samples processed with CB and o-xylene using a),c) spin-coating and b),d) blade coating, respectively.


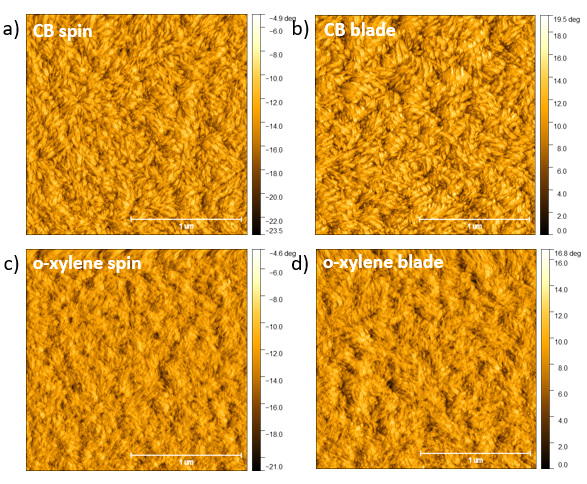


Figure S5. AFM phase images of samples processed with CB and o-xylene using a),c) spin-coating and b),d) blade coating, respectively.


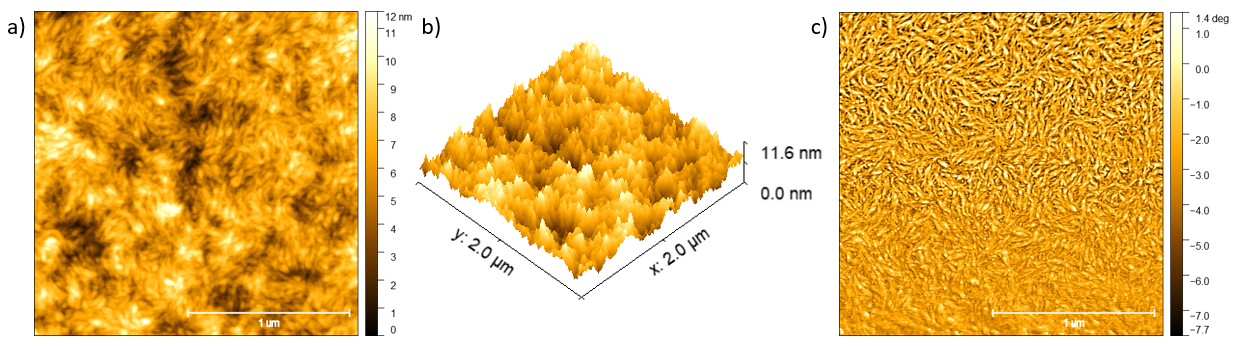


Figure S6. a) AFM topography, b) 3D topography and c) phase images of 100 nm slot-die coated active layers deposited with o-xylene.

Table S2: IP and OOP coherence lengths for all o-xylene devices, with different active layer thicknesses and fabrication techniques.

| Direction | Sample | Active Layer Thickness (nm) | Peak 1 |  | L_c_ (Å) from Peak 1 | Peak 2 |  |
| --- | --- | --- | --- | --- | --- | --- | --- |
|  |  |  | **q (Å^-1^)** | **d (Å)** |  | **q (Å^-1^)** | **d** |
| IP | spin | 100 | 0.30 | 21.0 | 37.9 |  |  |
|  | spin | 180 | 0.29 | 21.4 | 59.1 | 0.40 | 15.8 |
|  | blade | 115 | 0.29 | 21.6 | 55.9 |  |  |
|  | blade | 180 | 0.29 | 21.9 | 71.3 | 0.38 | 16.7 |
|  | slot-die | 115 | 0.29 | 21.8 | 59.5 | 0.41 | 15.3 |
|  | slot-die | 230 | 0.29 | 21.8 | 61.5 | 0.41 | 15.5 |
| OOP | spin | 100 | 0.28 | 22.4 | n/a |  |  |
|  | spin | 180 | 0.30 | 20.9 | 23.5 | 1.76 | 3.57 |
|  | blade | 115 | 0.29 | 21.7 | n/a |  |  |
|  | blade | 180 | 0.30 | 20.9 | 20.2 | 1.72 | 3.65 |
|  | slot-die | 115 | 0.31 | 20.3 | 24.1 | 1.80 | 3.49 |
|  | slot-die | 230 | 0.31 | 20.3 | 25.6 | 1.79 | 3.51 |
